# EEG-Based Brain-Computer Interface for Decoding Motor Imagery Tasks within the Same Hand Using Choi-Williams Time-Frequency Distribution

**DOI:** 10.3390/s17091937

**Published:** 2017-08-23

**Authors:** Rami Alazrai, Hisham Alwanni, Yara Baslan, Nasim Alnuman, Mohammad I. Daoud

**Affiliations:** 1 Department of Computer Engineering, School of Electrical Engineering and Information Technology, German Jordanian University, Amman 11180, Jordan; mohammad.aldaoud@gju.edu.jo; 2 Faculty of Engineering, University of Freiburg, Freiburg 79098, Germany; hisham.alwanni@pluto.uni-freiburg.de; 3 Department of Biomedical Engineering, School of Applied Medical Sciences, German Jordanian University, Amman 11180, Jordan; y.baslan@gju.edu.jo (Y.B.); nasim.alnuman@gju.edu.jo (N.A.)

**Keywords:** motor imagery, Choi-Williams time-frequency distribution, electroencephalography, time-frequency features, hierarchical classification, support vector machines, subject-independent analysis

## Abstract

This paper presents an EEG-based brain-computer interface system for classifying eleven motor imagery (MI) tasks within the same hand. The proposed system utilizes the Choi-Williams time-frequency distribution (CWD) to construct a time-frequency representation (TFR) of the EEG signals. The constructed TFR is used to extract five categories of time-frequency features (TFFs). The TFFs are processed using a hierarchical classification model to identify the MI task encapsulated within the EEG signals. To evaluate the performance of the proposed approach, EEG data were recorded for eighteen intact subjects and four amputated subjects while imagining to perform each of the eleven hand MI tasks. Two performance evaluation analyses, namely channel- and TFF-based analyses, are conducted to identify the best subset of EEG channels and the TFFs category, respectively, that enable the highest classification accuracy between the MI tasks. In each evaluation analysis, the hierarchical classification model is trained using two training procedures, namely subject-dependent and subject-independent procedures. These two training procedures quantify the capability of the proposed approach to capture both intra- and inter-personal variations in the EEG signals for different MI tasks within the same hand. The results demonstrate the efficacy of the approach for classifying the MI tasks within the same hand. In particular, the classification accuracies obtained for the intact and amputated subjects are as high as 88.8% and 90.2%, respectively, for the subject-dependent training procedure, and 80.8% and 87.8%, respectively, for the subject-independent training procedure. These results suggest the feasibility of applying the proposed approach to control dexterous prosthetic hands, which can be of great benefit for individuals suffering from hand amputations.

## 1. Introduction

Nowadays, many individuals are suffering from hand motor impairments due to strokes, hand amputations, and spinal cord injuries. Developing a system that can recover a significant part of the lost or disabled hand functionality is crucial to improve the quality of life of those individuals. Recently, we have witnessed substantial advancements in designing and developing wearable assistive devices, such as robotic prosthetic hands and exoskeletal orthotic hands. These assistive devices can be of great benefit for individuals who are cognitively intact and suffering from motor impairments. In particular, an individual with amputated hand can utilize a prosthetic hand to recover part of the missing hand functionality [1]. Moreover, an individual who had a stroke attack can utilize an exoskeletal orthotic hand to support his/her disabled hand [1]. In this vein, brain-computer interface (BCI) systems have been employed to provide alternative non-muscular communication pathways to assist people suffering from motor disabilities or living with lost limbs to interact with their surroundings [1,2,3].

BCI systems translate the neural signals of the human brain into control commands for peripheral and assistive devices, which in turn can improve the communication capabilities of the individuals who suffer from severe motor impairments. Several noninvasive neuroimaging modalities have been utilized in BCI systems, such as functional magnetic resonance imaging (fMRI) [4], electroencephalography (EEG) [5,6,7,8,9], and positron emission tomography (PET) [10,11]. Among these different neuroimaging modalities, EEG is considered the most commonly used modality in BCI systems. This can be attributed to several factors such as the high temporal resolution, relatively low cost, and high portability [12,13,14]. The use of EEG provides a measure of the electrical potentials generated at various locations of the brain in response to the execution or imagination of different movements [15].

Over the past two decades, motor imagery (MI) has been used to design EEG-based BCI systems that enable individuals with motor impairments to control various assistive devices, such as wheelchairs [16,17], prosthetic devices [18,19,20], and computers [1,21]. In fact, a MI task can be defined as a mental process in which an individual imagines himself/herself performing a specific action without real activation of the muscles [22]. During MI tasks, various regions in the brain are activated such as primary motor cortex (M1), primary and secondary sensory areas, pre-frontal areas, superior and inferior parietal lobules, and dorsal and ventral pre-motor cortices [15]. Therefore, the development of BCI systems that can effectively analyze brain signals and discriminate between different MI tasks to control neural prostheses devices has the potential to enhance the quality of life for people with severe motor disabilities.

Literature reveals that the vast majority of the existing MI EEG-based BCI systems were focused on differentiating between MI tasks that are associated with four different body parts [23,24,25,26,27], including feet, left hand, right hand, and tongue. Despite the relatively high classification accuracies attained for classifying MI tasks performed by different body parts, the discrimination between MI tasks within the same hand is considered challenging [6,7,8,9]. This can be attributed to three limitations associated with the EEG signals. First, the low spatial resolution of the EEG signals constrains the ability to discriminate between MI tasks of the same hand that activate similar and close areas in the brain [6]. In fact, this limitation becomes more pronounced when the MI tasks are associated with the same joint in the hand, such as wrist movements. Second, due to the volume conducted effect [28], EEG signals have a limited signal-to-noise ratio [6]. This in turn can drastically reduce the ability to discriminate between EEG signals of different dextrous MI tasks within the same hand, such as fingers- and wrist-related tasks. Third, the spectral characteristics of the EEG signals are time varying, or non-stationary. The non-stationary characteristics of EEG signals introduce large intra-trial variations for each subject and inter-personal variations between subjects, which increase the difficulty to discriminate between the EEG signals of MI tasks within the same hand. Therefore, traditional time-domain and frequency-domain representations, which are employing the time-invariance assumption, are considered inadequate to represent EEG signals [29,30,31,32].

Recently, a few studies have been reported to utilize EEG signals in order to discriminate between flexion/extension movements of the fingers [6,33] as well as several wrist movements [5,9], including flexion, extension, supination and pronation. The promising results reported in these studies demonstrate the possibility of utilizing EEG signals to discriminate between MI tasks within the same hand. Nonetheless, these studies have been conducted using EEG signals acquired from intact subjects, without exploring the capability of classifying MI tasks within the same hand using EEG signals that are acquired from individuals with hand amputations. Moreover, these studies, which explored a limited number of movements, have focused on decoding finger movements or wrist movements without attempting to discriminate between MI tasks associated with different parts of the hand.

The aim of the current study is to contribute to the ongoing research in the field of EEG signal analysis by introducing an EEG-based BCI system that employs an extracted set of time-frequency features (TFFs) to discriminate between eleven MI tasks within the same hand. In fact, we hypothesize that the use of time-frequency distribution (TFD) as joint time-frequency representation of EEG signals enables the extraction of salient TFFs that comprise discriminative information about different MI tasks within the same hand. The MI tasks considered in the present study range from basic wrist and fingers tasks, such as flexion/extension tasks, to complex hand tasks, such as functional grasping tasks. This diverse set of MI tasks makes the problem of classifying the MI tasks challenging, due to the substantial inter- and intra-personal variations of the EEG signals associated with different MI tasks.

In order to discriminate between the EEG signals associated with the eleven MI tasks, a sliding window approach is employed to decompose each EEG signal into overlapping segments. Then, we utilize the Choi-Williams TFD (CWD) to construct a time-frequency representation of the EEG segments that can describe the dynamic changes in the EEG signals during different MI tasks. Using the time-frequency representation, we extract five categories of TFFs, including log-amplitude-based category, amplitude-based category, statistical-based category, spectral-based category, and spectral entropy-based category. The extracted TFFs are utilized to construct a hierarchical classification model that classifies each EEG segment into one of the eleven MI tasks considered in this study. Specifically, the hierarchical classification model consists of four layers. The first layer classifies the EEG segments into rest or movement segments. In the second layer, the EEG segments that were identified as movement segments at the first layer are further classified into functional or basic wrist and finger movements. The third layer classifies the EEG segments that comprise functional movements into small diameter grasp, lateral grasp, and extension-type grasp, and the EEG segments that comprise basic wrist and fingers movements into wrist-related movements and finger-related movements. Finally, the fourth layer classifies the EEG segments that comprise wrist-related movements into wrist flexion/extension, wrist ulnar/radial, and the EEG segments that comprise finger-related movements into index flexion/extension, middle flexion/extension, ring flexion/extension, little flexion/extension, and thumb flexion/extension.

In order to evaluate the performance of the proposed approach, we have recorded EEG data for both intact and amputated subjects while imagining to perform the eleven hand MI tasks. Two performance evaluation analyses are conducted to evaluate the performance of the proposed TFD-based approach in identifying the eleven different MI tasks. The two performance evaluation analyses are: the channel-based performance evaluation analysis and the TFF-based performance evaluation analysis. These performance evaluation analyses quantify the effect of the utilized EEG channel locations and TFFs on the capability of the proposed system to decode different MI tasks within the same hand. Furthermore, within each evaluation analysis, the hierarchial classification model is trained using two different procedures, namely subject-dependent and subject-independent training procedures. These two training procedures measure the ability of the proposed TFD-based system to capture both intra- and inter-personal variations of the EEG signals for different MI tasks within the same hand. To the best of our knowledge, this is the first study that explores the use of TFD for classifying MI tasks within the same hand for both intact and amputated subjects.

The remainder of this paper is organized as follows: In Section 2, we describe the experimental procedure, the proposed TFD-based features, the classification model, and the evaluation procedures. The experimental results and discussion are presented in Section 3 and Section 4, respectively. Finally, the conclusion is provided in Section 5.

## 2. Materials and Methods

### 2.1. Subjects

The EEG dataset employed in the current study is composed of two databases, namely $DB_{1}$ and $DB_{2}$. $DB_{1}$ includes EEG signals acquired from eighteen intact subjects (6 females and 12 males, 4 left-handed and 14 right-handed) who volunteered to participate in the experiments. The mean ± standard deviation age of the subjects was 21.2±2.9 years. Furthermore, the subjects did not have any known neurological or neuromuscular disorders. In $DB_{2}$, four male subjects with upper limb amputation participated in the experiments. The mean ± standard deviation age of the subjects was 28.5±6.2 years. Table 1 provides characterization information about the amputations associated with the subjects who were recruited in $DB_{2}$. Before data acquisition, the experimental procedure of our study was explained in details to each subject and signed consent forms were collected from all subjects. The participants had the chance to withdraw from the study at anytime during the experimental procedure. Moreover, the experimental procedure was reviewed and approved by the Research Ethics Committee at the German Jordanian University.

### 2.2. Experimental Procedure

**Figure 1:**
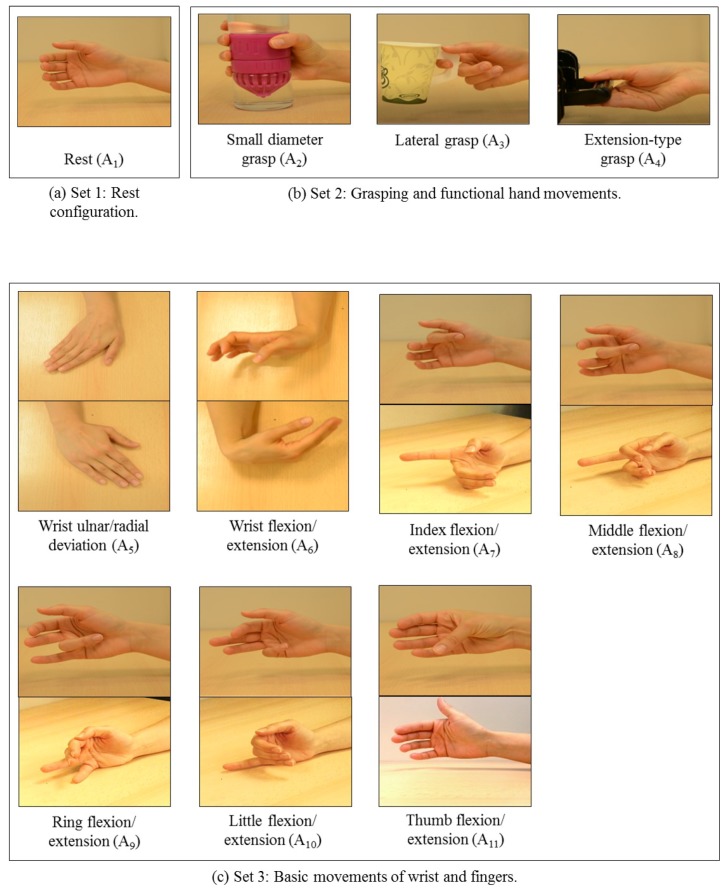
Sample images of the visual cues associated with the different HMITs investigated in this study.

The experimental procedure adopted in the current study is similar to the experimental procedures employed in several previous studies related to EEG-based MI tasks classification, such as [8,34,35,36]. In particular, each subject was seated on a comfortable upright chair at a distance of approximately 0.5 m from a computer monitor placed on top of a desk. During the experiments, the subjects were asked to comfortably rest their arms on the desk. Then, each subject was asked to imagine performing different hand tasks according to the displayed visual cues on the computer monitor. The visual cues associated with the hand tasks are shown in Figure 1. In this work, we consider three sets of hand motor imagery tasks (HMITs), namely set 1 (see Figure 1a), set 2 (see Figure 1b), and set 3 (see Figure 1c). Specifically, set 1 includes the rest configuration of the hand, which we denoted as $A_{1}$. Set 2 comprises grasping and functional movements of the hand, including the small diameter grasp ($A_{2}$), lateral grasp ($A_{3}$), and extension-type grasp ($A_{4}$). Finally, set 3 contains basic movements of the wrist and the fingers, including wrist ulnar/radial deviation ($A_{5}$), wrist flexion/extension ($A_{6}$), index finger flexion/extension ($A_{7}$), middle finger flexion/extension ($A_{8}$), ring finger flexion/extension ($A_{9}$), little finger flexion/extension ($A_{10}$), and thumb flexion/extension ($A_{11}$). The eleven HMITs that are comprised in the aforementioned three sets were selected to cover a wide range of the hand movements that are involved in activities of daily living (ADL) [35].

**Figure 2:**
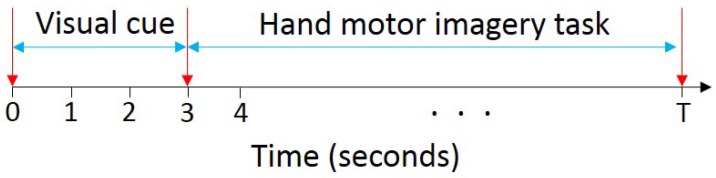
Experimental paradigm. A visual cue is displayed on the computer monitor for 3 s. After that, the visual cue disappears and a black screen is displayed on the monitor. During this period of time, the subject starts to imagine performing the movement that was specified by the visual cue until time *T* s. The value of *T* varies according to the complexity of the movement being imagined. In particular, for the movements in set 1 and set 3, *T* is equal to 10 s. For the extension-type grasp movement in set 2, *T* is equal to 12 s. Finally, for the small diameter grasp and lateral grasp movements in set 2, *T* is equal to 14 s.

The experimental procedure consists of a training phase and recording phase. In the training phase, each subject was asked to watch a set of videos displaying each of the movements depicted in Figure 1. Then, the subjects were asked to practice imagining themselves performing the displayed movements in order to become familiar with the experiment. During the recording phase, each subject was asked to relax his/her arms on the desk. Then, a visual cue was displayed on the computer monitor in front of the subject for 3 s. After that, the visual cue disappeared and a black screen was displayed on the monitor. The subject was asked to close his/her eyes when the screen turned black, and to start to imagine performing the movement that was specified by the visual cue until the experimenter prompted him/her that the recording was over. For $DB_{1}$, each subject was asked to imagine performing the eleven HMITs using his/her right hand. However, for $DB_{2}$, the subjects were asked to imagine performing each movement using the missing limb. The duration of the recorded EEG signals of the HMITs varies according to the complexity of the movement being imagined as depicted in Figure 2. In particular, for the movements in set 1 and set 3, the duration of each trial is equal to 10 s. For the extension-type grasp movement in set 2, the duration of each trial is equal to 12 s. Finally, for the small diameter grasp and lateral grasp movements in set 2, the duration of each trial is equal to 14 s. The average duration of the experiment for each subject was approximately 1.5 h. This time includes the subject preparation and the recording of 7 trials for each of the hand movements depicted in Figure 1.

### 2.3. EEG Data Acquisition and Preprocessing

**Figure 3:**
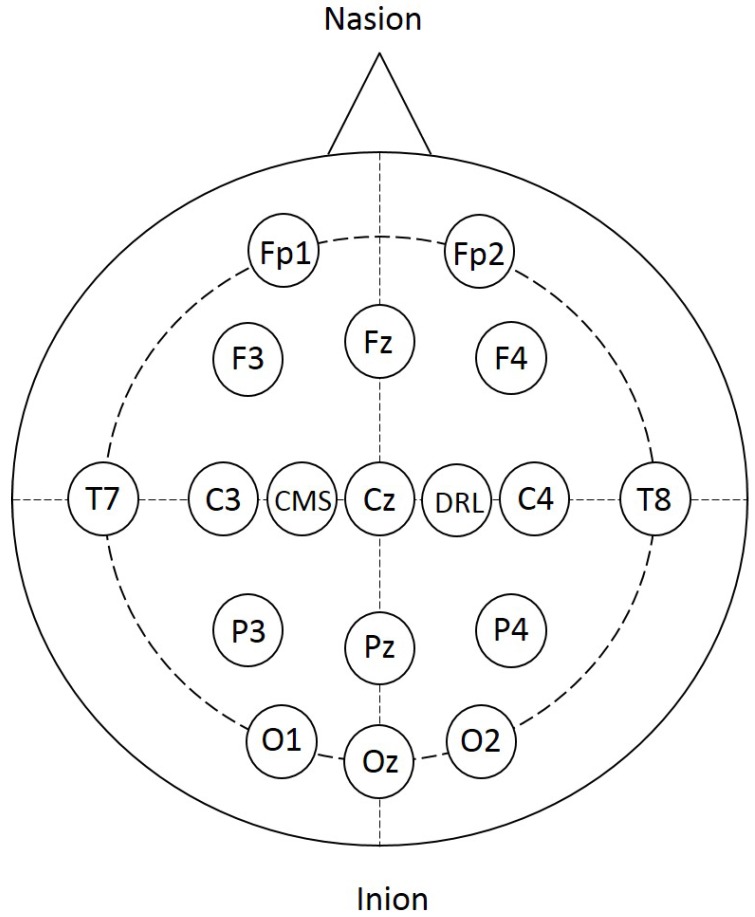
The positions of the EEG electrodes employed in this study arranged according to the 10–20 EEG system.

Raw EEG data was acquired using the Biosemi ActiveTwo EEG recording system (Biosemi B.V., Amsterdam, Netherlands). The Biosemi ActiveTwo system employs the 10–20 international EEG electrode placement system to localize 16 Ag/AgCl electrodes at the following locations: Fp1, Fp2, C3, C4, Cz, F3, F4, Fz, T7, T8, O1, O2, Oz, P3, P4, and Pz, referenced to the common mode sense (CMS)/ driven right leg (DRL) at C1/C2 locations for noise cancelation (see Figure 3). In this study, we consider four different groups of electrodes that cover different motor cortex related regions in the brain [9,36,37]. Table 2 shows the electrodes included within each group.

The EEG signals were acquired at a sampling frequency of 2048 Hz. The acquired signals were filtered using a band pass filter with a bandwidth of 0.5–35 Hz to reduce low-frequency noise [8,34] and ensure that the mu and beta rhythms, which are necessary for classifying EEG signals related to MI tasks, are within the bandwidth of the filtered EEG signals [8]. The filtered EEG signals were downsampled to 256 Hz to reduce the processing and storage requirements. In addition, EEGLAB toolbox [38] was utilized to remove the muscular and ocular artifacts from the acquired EEG signals using the automatic artifact rejection (AAR) toolbox [39].

### 2.4. Time-Frequency Representation of EEG Signals

The non-stationary nature of the EEG signals implies that the frequency contents of the EEG signals are rapidly changing over time [40]. This imposes the requirement of employing a time-frequency representation in order to effectively analyze the EEG signals. Indeed, recent studies on detecting seizure activities in EEG signals have indicated that utilizing joint time-frequency representations of EEG signals can significantly outperform traditional time-domain or frequency-domain representations [41,42]. This can be attributed to the fact that several key features of the EEG signals are encapsulated within either the time-domain or frequency-domain. Hence, the use of joint time-frequency representation has the potential to provide more discriminative features of EEG signals and can enhance the classification accuracy of MI tasks within the same hand.

**Figure 4:**
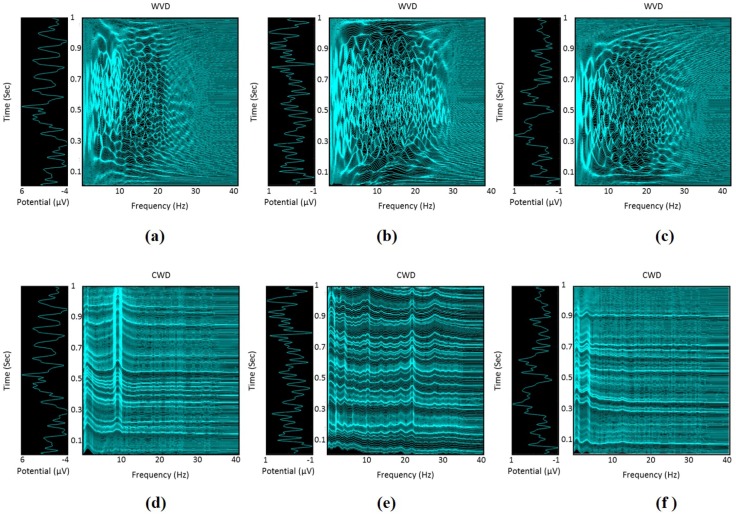
Illustration of the constructed time-frequency representation of EEG segments. The plots (**a**–**c**) represent the WVD of the EEG segments associated with rest, wrist flexion/extension, and lateral grasp, respectively. The plots (**d**–**f**) represent the CWD of the EEG segments associated with rest, wrist flexion/extension, and lateral grasp, respectively.

In this study, we propose a time-frequency representation for analyzing EEG signals that is based on computing the time-frequency distribution (TFD) of the EEG signals. Specifically, TFD can be viewed as a transformation that maps the EEG signals from the one-dimensional time-domain into a two-dimensional time-frequency plane (TFP), which allows capturing the spectral changes in the EEG signals occurring over time [43]. In order to compute the TFD of the acquired EEG signals, we segment the EEG signals of each channel using a sliding window of size W=256 samples and overlap size of O=128 samples. Each EEG segment is transformed into its analytic form to enhance the resolution of the TFP representation [44,45,46]. Specifically, the analytic signal of a real EEG segment x(t) can be defined as follows [43]:

(1) $$s_{x}\left(t\right)=x\left(t\right)+j\mathrm{HT}\left\{x\left(t\right)\right\},$$

where $s_{x}\left(t\right)$ is the analytic signal of x(t) and HT{·} is the Hilbert transform [47]. The time-frequency representation of the segment x(t) is carried out by computing the TFD of the analytic signal $s_{x}\left(t\right)$. In this vein, Cohen [44,45] provided a general formula to compute the TFD of an analytic signal, which can be applied to various types of distributions. In particular, the TFD of the analytic signal $s_{x}\left(t\right)$ can be computed as follows:

(2) $$\Gamma_{s}\left(t,f\right)=\int_{-\infty }^{\infty }\int_{-\infty }^{\infty }{\mathrm{AF}}_{s}\left(\phi ,\tau \right)\psi \left(\phi ,\tau \right)e^{-j2\pi f\tau -j2\pi t\phi }\partial_{\tau }\partial_{\phi },$$

where $\Gamma_{s}\left(t,f\right)$ is the TFD of the analytic signal $s_{x}\left(t\right)$ and ${\mathrm{AF}}_{s}\left(\phi ,\tau \right)$ is the ambiguity function of $s_{x}\left(t\right)$. The ambiguity function ${\mathrm{AF}}_{s}\left(\phi ,\tau \right)$ is defined as the Fourier transform of the auto-correlation function of s(t), which can be expressed as follows [44,45]:

(3) $${\mathrm{AF}}_{s}\left(\phi ,\tau \right)=\int_{-\infty }^{\infty }s_{x}\left(t+\frac{\tau }{2}\right)s_{x}^{*}\left(t-\frac{\tau }{2}\right)e^{j2\pi \phi t}\partial t,$$

where $s_{x}^{*}\left(\cdot \right)$ is the complex conjugate of $s_{x}\left(\cdot \right)$. In Equation (2), ψ(ϕ,τ) is the smoothing kernel function that defines the type of the TFD. In fact, various kernel functions can be employed to compute TFDs, where the design of these kernels depends on the information to be extracted from the TFP, the resolution in both time and frequency domains, and the ability to suppress the cross-terms generated from the bi-linearity of the TFDs [48]. When the kernel function is defined as ψ(ϕ,τ)=1, the generated TFD is called Wigner-Ville distribution (WVD) [49]. The WVD is a quadratic TFD that produces prevalent interference terms in the TFP, which are usually called cross-terms. The existence of cross-terms in the generated TFP increases the difficulty of interpreting the energy distribution in the TFP as a function of both time and frequency [43]. Therefore, in this study, we utilize the Choi-Williams distribution (CWD) [50] in order to minimize the cross-terms in the TFP. Unlike the WVD, the CWD employs an exponential kernel function to suppress the cross-term artifacts while maintaining a good resolution in the TFP [40,43]. The kernel function of the CWD can be expressed as follows [50]:

(4) $$\psi \left(\phi ,\tau \right)=\exp\left(-\frac{\phi^{2}\tau^{2}}{\gamma^{2}}\right),$$

where γ>0 is a parameter that controls the suppression of the cross-terms and its value is experimentally selected to be 0.5. Figure 4 shows the time-frequency representations computed for three EEG segments that represent three HMITs, namely rest, wrist flexion/extension, and lateral grasp. These time-frequency representations demonstrate the effect of utilizing the CWD on reducing the cross-terms in comparison with the WVD, which in turn enables a more distinguishable TFPs for differentiating HMITs. The dimensionality of the constructed time-frequency representation for each EEG segment is equal to W×N, where *W* and *N* represent the number of time-domain samples of s(t) and the number of frequency-domain samples, respectively. In this study, we have only used the CWD to compute the time-frequency representation of the EEG segments. In fact, the computation of the CWD is carried out using the HOSA toolbox [51], where the values of *W* and *N* are set to 256 and 512, respectively.

### 2.5. Time-Frequency Features

The constructed CWD-based time-frequency representation of each EEG segment has a 256×512 points. Therefore, to reduce the dimensionality of the constructed time-frequency representation, we extract a set of 12 time-frequency features (TFFs) from the CWD of each EEG segment. In this study, we group the extracted TFFs into five different categories, namely the log-amplitude-based category ($C_{1}$), amplitude-based category ($C_{2}$), statistical-based category ($C_{3}$), spectral-based category ($C_{4}$), and spectral entropy-based category ($C_{5}$). These categories are described as follows:

#### 2.5.1. Log-Amplitude-Based Category

In this category, we adopt and extend the concept of moment-related features presented in [34], in which MI tasks associated with different limbs were classified by computing spectral moment-related features extracted from the bispectrum of the EEG signals. Among the different spectral moment-related features, the sum of the logarithmic amplitudes of the bispectrum achieved promising classification results [34]. Thus, in this study, we compute a TFF ($TF_{1}$) that quantifies the sum of the logarithmic amplitudes of the CWD of an EEG segment. The feature $TF_{1}$ is defined as follows:

(5) $$TF_{1}=\sum_{t=1}^{W}\sum_{f=1}^{N}\log(|\Gamma_{s}\left(t,f\right)\left|\right),$$

where $\Gamma_{s}\left(t,f\right)$ is the CWD of the analytic signal $s_{x}\left(t\right)$.

#### 2.5.2. Amplitude-Based Category

In this category, we utilize the amplitudes of the points in the CWD to classify the EEG segments. In particular, we adopt three amplitude-based TFFs [42,52,53,54], including the median absolute deviation of the CWD ($TF_{2}$), the root mean square value of the CWD ($TF_{3}$), and the inter-quartile range of the CWD ($TF_{4}$). The features $TF_{2}$, $TF_{3}$, and $TF_{4}$ can be expressed as follows [42]:

(6) $$TF_{2}=\frac{1}{WN}\sum_{t=1}^{W}\sum_{f=1}^{N}|\Gamma_{s}\left(t,f\right)-\left(\frac{1}{WN}\sum_{t=1}^{W}\sum_{f=1}^{N}\Gamma_{s}\left(t,f\right)\right)|.$$

(7) $$TF_{3}=\sqrt{\frac{1}{WN}\left(\sum_{t=1}^{W}\sum_{f=1}^{N}\Gamma_{s}\left(t,f\right)\right)}.$$

(8) $$TF_{4}=\frac{1}{N}\sum_{f=1}^{N}\left(\Gamma_{s}\left(\frac{3(W+1)}{4},f\right)-\Gamma_{s}\left(\frac{\left(W+1\right)}{4},f\right)\right).$$

#### 2.5.3. Statistical-Based Category

This category of features consists of the mean ($TF_{5}$), variance ($TF_{6}$), skewness ($TF_{7}$), and kurtosis ($TF_{8}$) of the CWD computed for each EEG segment [42,43,48]. These features can be defined as follows:

(9) $$TF_{5}=\frac{1}{WN}\sum_{t=1}^{W}\sum_{f=1}^{N}\Gamma_{s}\left(t,f\right).$$

(10) $$TF_{6}=\frac{1}{WN}\sum_{t=1}^{W}\sum_{f=1}^{N}{\left(\Gamma_{s}\left(t,f\right)-TF_{5}\right)}^{2}.$$

(11) $$TF_{7}=\frac{1}{WN{\left(TF_{6}\right)}^{3/2}}\sum_{t=1}^{W}\sum_{f=1}^{N}{\left(\Gamma_{s}\left(t,f\right)-TF_{5}\right)}^{3}.$$

(12) $$TF_{8}=\frac{1}{WN{\left(TF_{6}\right)}^{2}}\sum_{t=1}^{W}\sum_{f=1}^{N}{\left(\Gamma_{s}\left(t,f\right)-TF_{5}\right)}^{4}.$$

#### 2.5.4. Spectral-Based Category

The features in this category are based on adapting some of the frequency-domain spectral features of the EEG signals to the time-frequency domain. In particular, we employ two spectral-based TFFs [31,43,48], namely the flatness of the CWD ($TF_{9}$) and the flux of the CWD ($TF_{10}$). These two TFFs are the time-frequency extension of the spectral flux and spectral flatness in the frequency domain [42,52,55]. The use of spectral-based TFFs enables the quantification of several spectral information of the EEG signals, which can be used to classify different HMITs. In particular, the flatness of the CWD provides a measure that describes the uniformity of the distribution of the signal energy in the TFP [48]. Moreover, the flux of the CWD quantifies the changing rate of the signal energy in the TFP [48]. In this study, the features $TF_{9}$ and $TF_{10}$ are defined as follows [48]:

(13) $$TF_{9}=WN\frac{\prod_{t=1}^{W}\prod_{f=1}^{N}\left(\right|\Gamma_{s}\left(t,f\right){\left|\right)}^{1/WN}}{\sum_{t=1}^{W}\sum_{f=1}^{N}\left(\right|\Gamma_{s}\left(t,f\right)\left|\right)}.$$

(14) $$TF_{10}=\sum_{t=1}^{W-l}\sum_{f=1}^{N-k}\left(\Gamma_{s}\left(t+l,f+k\right)-\Gamma_{s}\left(t,f\right)\right),l=k=1.$$

#### 2.5.5. Spectral Entropy-Based Category

This category comprises two TFFs, namely the normalized Renyi entropy of the CWD ($TF_{11}$) and the energy concentration of the CWD ($TF_{12}$) [31]. The normalized Renyi entropy of the CWD measures the regularity of the distribution of the signal energy in the TFP. In fact, the EEG signals that have a uniformly distributed energy in the TFP tend to have a larger values of $TF_{11}$, while the signals that have energy concentrated within specific regions in the TFP tend to have smaller values of $TF_{11}$ [43,56,57]. The energy concentration of the CWD measures the spread of the energy in the TFP. Specifically, EEG signals that have broadly distributed energy across the TFP tend to have a larger values of $TF_{12}$, while signals that have energy concentrated within specific areas in the TFP tend to have smaller values of $TF_{12}$ [58]. In this study, the features $TF_{11}$ and $TF_{12}$ are defined as follows:

(15) $$TF_{11}=-\frac{1}{2}\log_{2}\sum_{t=1}^{W}\sum_{f=1}^{N}{\left(\frac{\Gamma_{s}\left(t,f\right)}{WN(TF_{5})}\right)}^{2}.$$

(16) $$TF_{12}={\left(\sum_{t=1}^{W}\sum_{f=1}^{N}\sqrt{|\Gamma_{s}\left(t,f\right)|}\right)}^{2}.$$

### 2.6. Classification of HMITs

As indicated by Edelman et al. [9], the EEG signals are characterized by low spatial resolution in the motor cortex regions. Hence, classifying EEG segments that encapsulate different MI tasks is considered challenging, particularly when these tasks are within the same hand. Another challenge is the variability in the duration of the HMITs, in which the length of each MI task depends on the complexity of the movement being imagined. Hence, the number of samples associated with different HMITs can vary significantly, which leads to unbalanced data samples across different HMITs. Therefore, direct application of a multi-class classifier for classifying the EEG segments into different MI movements within the same hand might lead to limited recognition accuracy [59,60].

**Figure 5:**
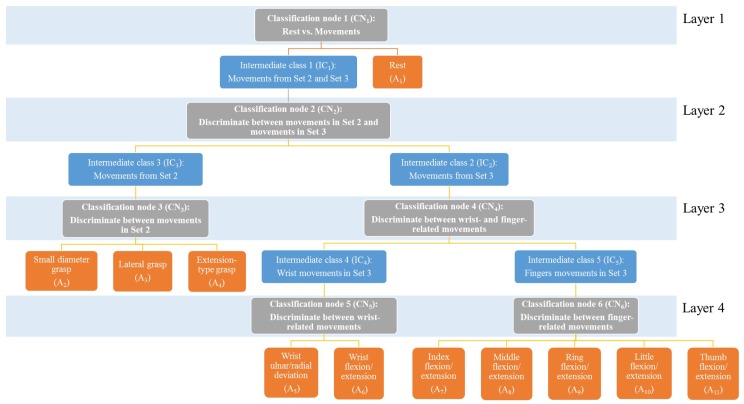
Illustration of the structure of the proposed four-layer hierarchical classification model. Nodes in gray color represent the classification nodes in each layer. Orange nodes represent the eleven classes of the HMITs in our study. Blue nodes represent an intermediate classes of EEG segments.

To address this limitation, we propose a four-layer hierarchical classification model to classify each EEG segment into one of the eleven HMITs considered in our study. The four-layers in our classification model convert the original complex classification task (i.e., classifying an EEG segment into one of the eleven HMITs) into a sequence of simpler classification tasks that are performed at each layer. In particular, the first layer consists of a classification node, namely $CN_{1}$, that classifies EEG segments into rest segments ($A_{1}$) and movement segments ($IC_{1}$), where movement segments are EEG segments that can comprise HMITs from set 2 or set 3 in our collected dataset. Then, the EEG segments of class $IC_{1}$ are passed on to the second layer to identify whether the movement in each EEG segment belongs to set 2 or set 3. Specifically, the second layer consists of a classification node, denoted as $CN_{2}$, that classifies each EEG segment of class $IC_{1}$ into a movement segment that comprises HMIT from set 2 ($IC_{2}$) or a movement segment that comprises HMIT from set 3 ($IC_{3}$). Then, the EEG segments of classes $IC_{2}$ and $IC_{3}$ are passed on to layer 3, which consists of two classification nodes, namely $CN_{3}$ and $CN_{4}$. At the third layer, $CN_{3}$ classifies the EEG segments of class $IC_{2}$ into one of the three HMITs comprised in set 2, namely small diameter grasping ($A_{2}$), lateral grasping ($A_{3}$), and extension-type grasp ($A_{4}$). Similarly, $CN_{4}$ classifies EEG segments of class $IC_{3}$ into wrist-related HMITs ($IC_{4}$) or finger-related HMITs movements ($IC_{5}$). Finally, the EEG segments of classes $IC_{4}$ and $IC_{5}$ are passed on to layer 4, which consists of two classification nodes, namely $CN_{5}$ and $CN_{6}$. At the fourth layer, $CN_{5}$ classifies the EEG segments of class $IC_{4}$ into wrist ulnar/radial deviation ($A_{5}$) and wrist flexion/extension ($A_{6}$). Similarly, $CN_{6}$ classifies the EEG segments of class $IC_{5}$ into one of the five finger-related HMITs comprised in set 3, namely index finger flexion/extension ($A_{7}$), middle finger flexion/extension ($A_{8}$), ring finger flexion/extension ($A_{9}$), little finger flexion/extension ($A_{10}$), and thumb flexion/extension ($A_{11}$). Figure 5 provides a schematic diagram of the proposed four-layer hierarchical classification model.

In this study, the input to the hierarchical classification model is a feature vector that consists of the TFFs extracted from the CWD computed for an EEG segment. Moreover, the classification nodes in the four layers are implemented using support vector machine (SVM) classifiers with radial basis function (RBF) kernel [61,62]. Previous studies have shown that utilizing SVM classifiers with RBF kernel can be more effective than generative models for supervised learning problems [63]. Moreover, using the SVM classifier with RBF kernel can achieve a better performance and generalization compared with the other state-of-the-art classifiers, such as Naive Bayes, k-nearest neighbors (k-NN), and neural networks [31,64]. Therefore, we realize the classification nodes $CN_{1},CN_{2},CN_{4}$ and $CN_{5}$ using binary SVM classifiers. The classification nodes $CN_{3}$ and $CN_{6}$ are realized using multi-class SVM classifiers. The multi-class SVM classifiers are implemented using a one-against-one scheme [65,66], in which we construct n(n−1)/2 binary SVM classifiers for each classification node, where *n* is the number of classes. In particular, for $CN_{3}$, the number of classes is three, including $A_{2}$, $A_{3}$ and $A_{4}$, whereas the number of classes for $CN_{6}$ is five, including $A_{7}$, $A_{8}$, $A_{9}$, $A_{10}$ and $A_{11}$.

The performance of the SVM classifier with RBF kernel depends on the selected values of the RBF kernel parameter (σ) and the regularization parameter (C>0). To tune these two parameters, we perform a grid-based search [66] along two directions to determine the values of σ and *C* for each classification node. In the first direction, we vary the value of the parameter σ, while in the second direction we vary the value of the parameter *C*. Then, the best SVM model is selected such that its parameters maximize the average classification accuracy.

### 2.7. Performance Evaluation Procedures and Metrics

The acquired EEG signals of the intact subjects ($DB_{1}$) and amputated subjects ($DB_{2}$) are used to perform two types of performance evaluation analyses, namely channel-based analysis and TFF-based analysis. In the channel-based analysis, we study the effect of selecting different groups of EEG channels on the accuracy of classifying HMITs. In particular, we evaluate the performance of our proposed approach based on its ability to classify the feature vectors extracted from the EEG channels comprised within each of the four channel groups ($G_{1}$, $G_{2}$, $G_{3}$ and $G_{4}$) presented in Table 2. For the TFF-based analysis, we explore the effect of using each of the five categories of TFFs, namely $C_{1}$, $C_{2}$, $C_{3}$, $C_{4}$ and $C_{5}$, on the accuracy of classifying HMITs. For each performance evaluation analysis, we measure the performance of the proposed approach using standard performance evaluation metrics, including the precision (PRC), recall (RCL), $F_{1}$-score, and accuracy (ACC), which are defined as follows [67,68]:

(17) $$PRC=\frac{TP}{\left(TP+FP\right)}\times 100\%,$$

(18) $$RCL=\frac{TP}{\left(TP+FN\right)}\times 100\%,$$

(19) $$F_{1}-score=2\frac{\left(PRC*RCL\right)}{\left(PRC+RCL\right)}\times 100\%,$$

(20) $$ACC=\frac{\left(TP+TN\right)}{\left(TP+TN+FP+FN\right)}\times 100\%,$$

where TP, TN, FP and FN represent the numbers of true positive cases, true negative cases, false positive cases, and false negative cases, respectively. These performance evaluation metrics are obtained using two types of training procedures, including subject-dependent training procedure (SDTP) and subject-independent training procedure (SITP). In the SDTP, we employ a ten-fold cross-validation procedure [8,69] to construct a hierarchical classification model for each subject, as described in Section 2.6. In particular, we randomly divide the feature vectors associated with the HMITs performed by each subject into 10 folds. Nine folds are used to train the classification nodes at each layer in the constructed classification model, while the remaining fold is used for testing. This procedure is repeated for ten times, and the overall performance of each classification node is computed by averaging the results obtained from each repetition. The SDTP measures the ability of the proposed approach to capture the intra-personal variations of the performed HMITs. For the SITP, we employ a leave-one-subject-out cross-validation (LOSO-CV) procedure to evaluate the performance of the proposed approach [31]. This procedure is based on constructing a single hierarchical classification model for all the subjects in each database. Then, the classification nodes at each layer of the constructed classification model are trained using the feature vectors extracted from all subjects except one subject. The feature vectors of the subject that was excluded from the training are used for testing. This procedure is repeated for each subject to guarantee that the feature vectors of each subject are used for testing, and the overall performance is computed by averaging the results obtained from all repetitions. The SITP quantifies the ability of the proposed approach to capture the inter-personal variations of the performed HMITs.

## 3. Experimental Results

In this section, we present the performance evaluation results of the proposed approach for the channel-based and TFF-based evaluation analyses obtained for the intact and amputated subjects using the SDTP and SITP.

### 3.1. Evaluation Results of the Intact Subjects ($DB_{1}$)

In this section, we evaluate the performance of the proposed approach based on $DB_{1}$ that includes the EEG signals of the intact subjects. In particular, we provide the performance evaluation results of the channel-based analysis and the TFF-based analysis obtained using the SDTP and SITP.

#### 3.1.1. Results of the Channel-Based Analysis

Table 3 provides the average PRC, RCL, $F_{1}$-score, and ACC of the classification nodes for each group of EEG channels, computed using the SDTP and SITP. In particular, for the SDTP, we extract the twelve TFFs ($TF_{1}$–$TF_{12}$) from the EEG signals in each group of EEG channels. Then, for each subject, we construct four hierarchical classification models using the TFFs extracted from the four groups of EEG channels. The classification nodes in each classification model are trained and tested using the ten-fold cross-validation procedure, which is described in Section 2.7. Finally, for each classification node, we report the average values of the PRC, RCL, $F_{1}$-score, and ACC metrics computed over the eighteen subjects in $DB_{1}$. For SITP, we utilize the twelve TFFs extracted from each group of EEG channels to construct a single hierarchical classification model for all the subjects in $DB_{1}$. The classification nodes in the constructed classification model are trained and tested using the LOSO-CV procedure, described in Section 2.7. Finally, for each classification node, we present the average values of the PRC, RCL, $F_{1}$-score, and ACC metrics computed over the repetitions of the LOSO-CV procedure.

In Table 3, the results obtained using SDTP show that the classification nodes achieved an average PRC, RCL, $F_{1}$-score, and ACC values that are higher than 70% using the various groups of EEG channels. In fact, the lowest PRC, RCL, $F_{1}$-score, and ACC values of 73.2%, 71.4%, 72.3% and 73.8%, respectively, are obtained using the TFFs extracted from the EEG channels of $G_{3}$. Moreover, the highest PRC, RCL, $F_{1}$-score, and ACC values of 84.4%, 82.9%, 83.6% and 84.6%, respectively, are achieved using the TFFs extracted from the EEG channels of $G_{1}$. On the other hand, the results obtained based on the SITP show that the classification nodes achieved an average PRC, RCL, $F_{1}$-score, and ACC values that are higher than 52% using the different groups of EEG channels, with the lowest PRC, RCL, $F_{1}$-score, and ACC of 57.4%, 53.0%, 54.9% and 58.7%, respectively, obtained using the TFFs extracted from the EEG channels of $G_{3}$ and the highest PRC, RCL, $F_{1}$-score, and ACC of 64.2%, 62.2%, 63.2% and 67.8%, respectively, obtained using the TFFs extracted from the EEG channels of $G_{1}$. In fact, the results obtained using both SDTP and SITP are well above the average random classification accuracy, which is defined as the the reciprocal of the number of classes, i.e. 9.1%.

To compare between the performance of the proposed approach using each group of EEG channels, we have conducted paired t-tests with significance level of 0.05 to compare the accuracies of the classification nodes obtained using the TFFs extracted from the EEG channels of $G_{1}$ with the accuracies of the classification nodes achieved based on the TFFs extracted from the other three groups of EEG channels. For the SDTP, the computed *p* values for $G_{1}$ versus $G_{2}$, $G_{1}$ versus $G_{3}$ and $G_{1}$ versus $G_{4}$ were 0.0007, 0.004 and 0.0014, respectively. Similarly, for the SITP, the computed *p* values for $G_{1}$ versus $G_{2}$, $G_{1}$ versus $G_{3}$ and $G_{1}$ versus $G_{4}$ were 0.0141, 0.0101 and 0.0072, respectively. The calculated *p* values, for both SDTP and SITP, demonstrate that the performance of the classification nodes achieved based on the TFFs extracted from the EEG channels of $G_{1}$ outperforms significantly the performance of the classification nodes obtained using the TFFs of the other three groups.

#### 3.1.2. Results of the TFF-Based Analysis

Table 4 provides the average PRC, RCL, $F_{1}$-score, and ACC of the classification nodes computed for each of the five categories of TFFs using both SDTP and SITP. The results presented in Table 4 are based on the TFFs extracted from the EEG channels of $G_{1}$. The selection of $G_{1}$ is based on the results of the channel-based analysis, described in the previous subsection, in which the classification nodes achieved the best performance when the TFFs were extracted from the EEG channels of $G_{1}$ in both SDTP and SITP. More specifically, for the SDTP, we construct five hierarchical classification models for each subject. Each classification model is constructed using the TFFs comprised within one of the five categories of TFFs. The classification nodes in each classification model are trained and tested using the ten-fold cross-validation procedure, as described in Section 2.7. The reported results of each classification node are the average values of PRC, RCL, $F_{1}$-score, and ACC computed over the eighteen subjects in $DB_{1}$. For the SITP, we utilize the TFFs extracted from each of the five categories of TFFs to construct five hierarchical classification models for all subjects in $DB_{1}$. In particular, each classification model utilizes the TFFs of one of the five categories extracted from the EEG signals of all subjects. The classification nodes in the constructed classification models are trained and tested using the LOSO-CV procedure, as described in Section 2.7. Finally, for each classification node, we present the average values of PRC, RCL, $F_{1}$-score, and ACC computed over the repetitions of the LOSO-CV procedure.

In Table 4, the obtained results based on the SDTP indicate that the classification nodes achieved average PRC, RCL, $F_{1}$-score, and ACC values that are higher than 72% using the different categories of TFFs, with the lowest PRC, RCL, $F_{1}$-score, and ACC values of 74.4%, 72.9%, 73.6% and 75.2%, respectively, obtained using the TFFs of the statistical-based category and the highest PRC, RCL, $F_{1}$-score, and ACC values of 88.1%, 86.7%, 87.4% and 88.8%, respectively, achieved using the TFFs of the log-amplitude-based category. On the other hand, the obtained results based on the SITP show that the classification nodes achieved average PRC, RCL, $F_{1}$-score and ACC values that are higher than 51% using the different categories of TFFs, with the lowest PRC, RCL, $F_{1}$-score, and ACC values of 55.4%, 51.3%, 53.2% and 55.4%, respectively, obtained using the TFFs of the statistical-based category and the highest PRC, RCL, $F_{1}$-score and ACC values of 81.3%, 79.5%, 80.4% and 80.8%, respectively, obtained using the TFFs of the log-amplitude-based category. The results of both the SDTP and the SITP are well above the average random classification accuracy, which is equal to 9.1%.

To compare the performance of the proposed approach obtained using each of the five TFFs categories, we have conducted paired t-tests with significance level of 0.05 to compare the obtained accuracies of the classification nodes based on the TFF of $C_{1}$ with the accuracies of the classification nodes based on the TFFs of the other four categories. For the SDTP, the computed *p* values for $C_{1}$ versus $C_{2}$, $C_{1}$ versus $C_{3}$, $C_{1}$ versus $C_{4}$, and $C_{1}$ versus $C_{5}$ are 0.0042, 0.0015, 0.0032 and 0.0012, respectively. Similarly, for the SITP, the computed *p* values for $C_{1}$ versus $C_{2}$, $C_{1}$ versus $C_{3}$, $C_{1}$ versus $C_{4}$, and $C_{1}$ versus $C_{5}$ are 0.00071, 0.0005, 0.0019, and 0.0074, respectively. The calculated *p* values, for both SDTP and SITP, indicate that the performance of the classification nodes based on the TFF of $C_{1}$ outperforms significantly the performance of the classification nodes obtained using the TFFs of the other four categories.

**Figure 6:**
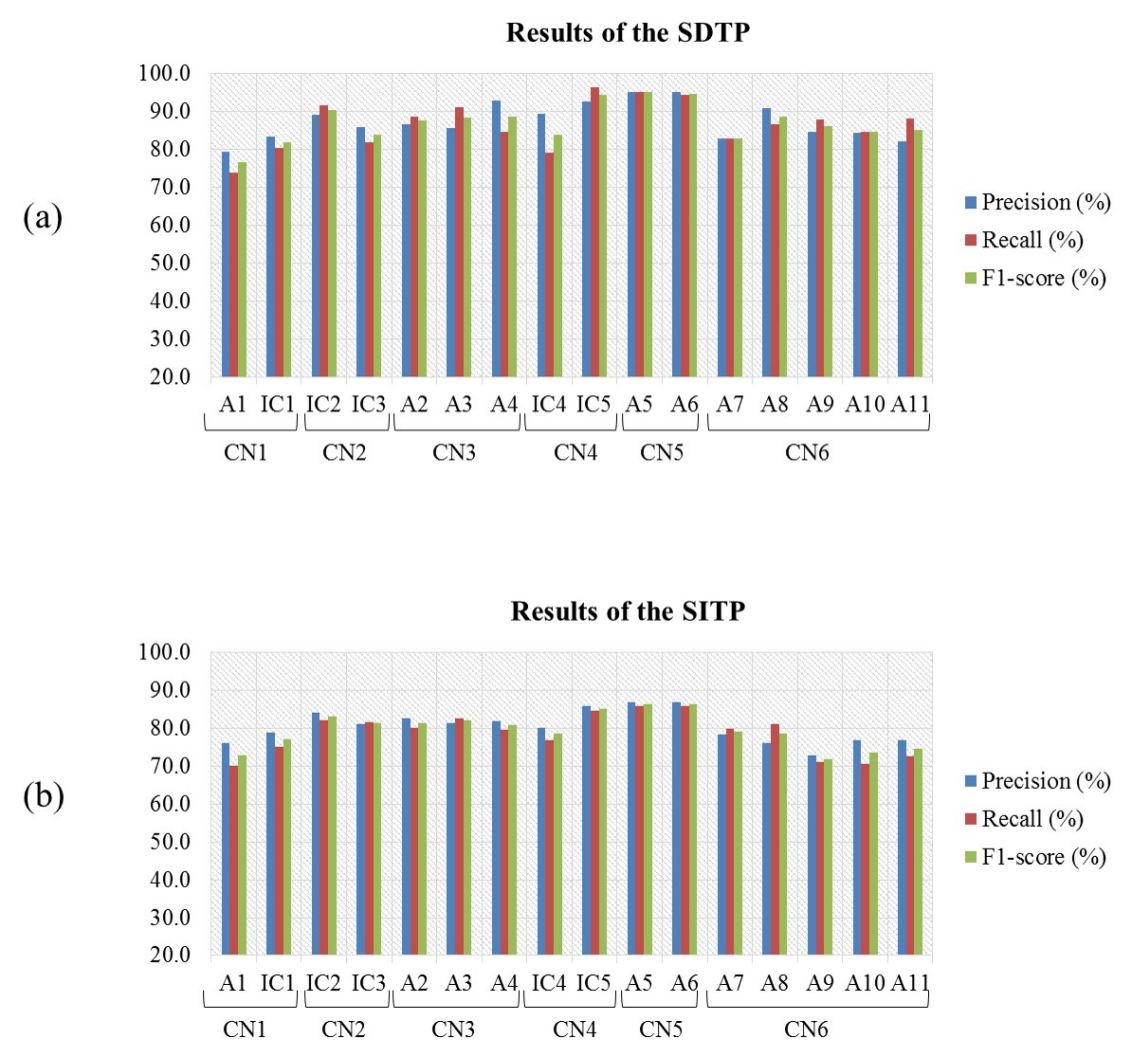
Average PRC, RCL, $F_{1}$-score values of each of the eleven HMITs and the five intermediate classes for the intact subjects obtained based on the TFF of $C_{1}$ using (**a**) SDTP and (**b**) SITP.

Figure 6 shows the average PRC, RCL, and $F_{1}$-score values obtained by the classification nodes ($CN_{1}$–$CN_{6}$) in terms of their ability to classify the eleven HMITs ($A_{1}$–$A_{11}$) and five intermediate classes ($IC_{1}$–$IC_{5}$) based on the TFF of $C_{1}$ for both SDTP and SITP.

### 3.2. Evaluation Results of the Amputated Subjects ($DB_{2}$)

In this section, we evaluate the performance of the proposed approach based on the acquired EEG signals of the amputated subjects recruited in $DB_{2}$. In particular, we provide the performance evaluation results of the channel-based analysis and the TFF-based analysis obtained using the SDTP and the SITP.

#### 3.2.1. Results of the Channel-Based Analysis

Table 5 provides the average PRC, RCL, $F_{1}$-score, and ACC of the classification nodes for each group of EEG channels, computed using the SDTP and SITP. In particular, for the SDTP, we extract twelve TFFs ($TF_{1}$–$TF_{12}$) from the EEG signals in each group of EEG channels. Then, for each subject, we construct four hierarchical classification models using the TFFs extracted from the four groups of EEG channels. The classification nodes in each classification model are trained and tested using the ten-fold cross-validation procedure. Finally, for each classification node, we report the average values of PRC, RCL, $F_{1}$-score, and ACC that are computed for the four subjects in $DB_{2}$. For SITP, we utilize the twelve TFFs extracted from each group of EEG channels to construct a single hierarchical classification model for all subjects in $DB_{2}$. The classification nodes in the constructed classification model are trained and tested using the LOSO-CV procedure. Finally, for each classification node, we present the average values of PRC, RCL, $F_{1}$-score, and ACC that are computed over the four repetitions of the LOSO-CV procedure.

In Table 5, the SDTP results indicate that the classification nodes achieved average PRC, RCL, $F_{1}$-score, and ACC values that are higher than 79% using the different groups of EEG channels, with the lowest PRC, RCL, $F_{1}$-score, and ACC values of 80.3%, 79.3%, 79.8% and 80.2%, respectively, obtained using the TFFs extracted from the EEG channels of $G_{2}$ and the highest PRC, RCL, $F_{1}$-score, and ACC values of 84.8%, 83.5%, 84.1% and 84.8%, respectively, achieved using the TFFs extracted from the EEG channels of $G_{1}$. On the other hand, the results obtained based on the SITP show that the classification nodes achieved average PRC, RCL, $F_{1}$-score, and ACC values that are higher than 61% using the different groups of EEG channels, with the lowest PRC, RCL, $F_{1}$-score, and ACC of 63.4%, 61.3%, 62.3% and 63.6%, respectively, obtained using the TFFs extracted from the EEG channels of $G_{2}$ and the highest PRC, RCL, $F_{1}$-score, and ACC of 73.0%, 72.0%, 72.5% and 73.9%, respectively, obtained using the TFFs extracted from the EEG channels of $G_{1}$. The results reported for both the SDTP and the SITP are well above the average random classification accuracy, which is equal to 9.1%.

To evaluate the performance of the proposed approach using each group of EEG channels, we have conducted paired t-tests with significance level of 0.05 to compare the accuracies obtained using the TFFs extracted from the EEG channels of $G_{1}$ with the accuracies of the classification nodes based on the TFFs extracted from the other three groups of EEG channels. For the SDTP, the computed *p* values for $G_{1}$ versus $G_{2}$, $G_{1}$ versus $G_{3}$ and $G_{1}$ versus $G_{4}$ are 0.0037, 0.0238 and 0.0084, respectively. Similarly, for the SITP, the *p* values for $G_{1}$ versus $G_{2}$, $G_{1}$ versus $G_{3}$ and $G_{1}$ versus $G_{4}$ are 0.0108, 0.0054 and 0.008, respectively. The calculated *p* values, for both SDTP and SITP, demonstrate that the performance of the classification nodes based on the TFFs that are extracted from the EEG channels of $G_{1}$ outperforms significantly the performance of the classification nodes obtained using the TFFs of the other three groups.

#### 3.2.2. Results of the TFF-Based Analysis

Table 6 provides the average PRC, RCL, $F_{1}$-score, and ACC of the classification nodes computed for each of the five categories of TFFs using both SDTP and SITP. The results presented in Table 6 are based on the TFFs extracted from the EEG channels of $G_{1}$. The selection of $G_{1}$ is based on the results of the channel-based analysis, described in the previous subsection, in which the classification nodes achieved their best performance when the TFFs were extracted from the EEG channels of $G_{1}$ in both SDTP and SITP. More specifically, for the SDTP, we construct five hierarchical classification models for each subject. Each classification model is constructed using the TFFs comprised within one of the five categories of TFFs. The classification nodes in each classification model are trained and tested using the ten-fold cross-validation procedure. The results reported for each classification node include the average values of the PRC, RCL, $F_{1}$-score, and ACC computed over the four subjects in $DB_{2}$. For the SITP, we utilize the TFFs extracted from each of the five categories of TFFs to construct five hierarchical classification model for all subjects in $DB_{2}$. In particular, each classification model utilizes the TFFs of one of the five categories extracted from the EEG signals of all subjects. The classification nodes in the constructed classification models are trained and tested using the LOSO-CV procedure. Finally, for each classification node, we present the average values of the PRC, RCL, $F_{1}$-score, and ACC computed over the four repetitions of the LOSO-CV procedure.

The SDTP results reported in Table 6 indicate that the classification nodes achieved average PRC, RCL, $F_{1}$-score and ACC values that are higher than 72% using the different categories of TFFs, with the lowest PRC, RCL, $F_{1}$-score and ACC values of 73.5%, 72.6%, 73.1% and 74.9%, respectively, obtained using the TFFs of the statistical-based category and the highest PRC, RCL, $F_{1}$-score and ACC values of 90.5%, 89.5%, 90.0% and 90.2%, respectively, obtained using the TFFs of the log-amplitude-based category. On the other hand, the obtained results based on the SITP show that the classification nodes achieved average PRC, RCL, $F_{1}$-score and ACC values thar are higher than 57% using the different categories of TFFs, with the lowest PRC, RCL, $F_{1}$-score and ACC of 60.0%, 57.4%, 58.7% and 61.0%, respectively, obtained using the TFFs of the statistical-based category and the highest PRC, RCL, $F_{1}$-score and ACC of 87.9%, 86.4%, 87.1% and 87.8%, respectively, obtained using the TFFs of the log-amplitude-based category. The results achieved using both the SDTP and the SITP are well above the average random classification accuracy.

To evaluate the performance of the proposed approach obtained using the five categories of TFFs, we have conducted paired t-tests with significance level of 0.05 to compare the accuracies of the classification nodes obtained using the TFF of $C_{1}$ with the accuracies of the classification nodes based on the TFFs of the other four categories. For the SDTP, the *p* values computed for $C_{1}$ versus $C_{2}$, $C_{1}$ versus $C_{3}$, $C_{1}$ versus $C_{4}$ and $C_{1}$ versus $C_{5}$ are 0.0098, 0.005, 0.0104 and 0.0084, respectively. Similarly, for the SITP, the *p* values computed for $C_{1}$ versus $C_{2}$, $C_{1}$ versus $C_{3}$, $C_{1}$ versus $C_{4}$ and $C_{1}$ versus $C_{5}$ are 0.146, 0.0063, 0.0067 and 0.0089, respectively. The *p* values calculated for the SDTP and SITP indicate that the performance of the classification nodes based on the TFF of $C_{1}$ outperforms significantly the performance of the classification nodes obtained using the TFFs of the other four categories.

**Figure 7:**
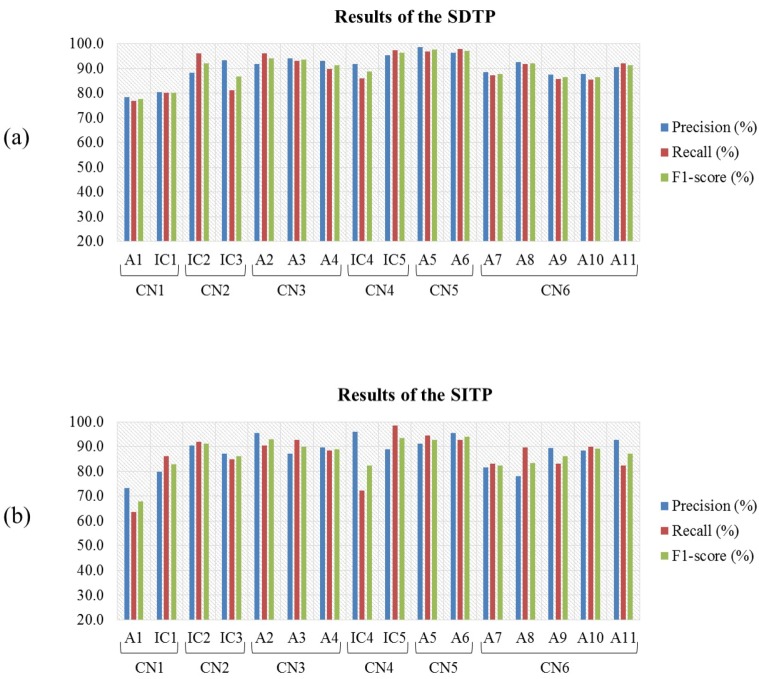
Average PRC, RCL, $F_{1}$-score values of each of the eleven HMITs and the five intermediate classes for the amputated subjects obtained based on the TFF of $C_{1}$ using (**a**) SDTP and (**b**) SITP.

Figure 7 shows the average PRC, RCL, and $F_{1}$-score values obtained by the classification nodes ($CN_{1}$–$CN_{6}$) in terms of their ability to classify the eleven HMITs ($A_{1}$–$A_{11}$) and five intermediate classes ($IC_{1}$–$IC_{5}$) based on the TFF of $C_{1}$ for both SDTP and SITP.

## 4. Discussion

This study aims to investigate the feasibility of using the CWD, which enables the extraction of TFFs from the EEG signals, along with a hierarchical classification model to discriminate between eleven HMITs, including: rest, basic finger and wrist movements, and grasping and functional movements. The results demonstrate that our proposed approach can classify the eleven HMITs and achieve promising performance in both subject-dependent and subject-independent evaluation scenarios.

### 4.1. Channel-Based Analyses

In order to study the effect of the utilized different EEG channels on the capability of the proposed approach to classify the eleven HMITs, channel-based analyses were carried out using four groups of EEG channels that overlay the motor cortex regions in the brain. The results of the channel-based analyses for both intact and amputated subjects, which are provided in Table 3 and Table 5 for both SDTP and SITP, indicate that our proposed approach achieved higher classification accuracies using the TFFs extracted from the EEG channels of $G_{1}$ compared to the performance obtained using the TFFs extracted from the other three groups of EEG channels. In fact, $G_{1}$ comprises EEG channels that cover various motor cortex regions on both sides of the brain, including: midline region, left and right fronto-central regions, left and right centro-parietal regions, and left and right temporal lobe regions, while the other three groups of EEG channels cover only subsets of the regions overlayed by the EEG channels of $G_{1}$. Hence, the results reported in Table 3 and Table 5 suggest that the electrical activities generated during different MI tasks within the motor cortex regions are propagating to various other regions in the brain. This might be attributed to the volume conductor effect on the EEG signals [28], in which the electrical activities generated within small cortical region are spatially propagated to other regions in the brain, and consequently recorded by the sparsely distributed electrodes on the scalp [6,7,28,70,71].

### 4.2. TFF-Based Analyses

The TFF-based analyses aimed to investigate the effect of utilizing different categories of TFFs on the classification accuracy of the eleven HMITs. Table 4 and Table 6 show that the best performance of our proposed approach was achieved using the TFF of $C_{1}$, which outperforms significantly the other four categories of TFFs, for both SDTP and SITP. Moreover, the results indicate that the performance of the proposed approach achieved higher performance using the TFFs of $C_{4}$ compared to the TFFs of $C_{2}$, $C_{3}$, and $C_{5}$, for both SDTP and SITP. On the other hand, the results obtained based on the TFFs of $C_{2}$, $C_{3}$, and $C_{5}$ vary depending on the training procedure and the EEG database used in the analysis. In addition, the results in Table 3 and Table 4 indicate that the performance of the hierarchical classification framework has been increased significantly when the TFF of $C_{1}$ was utilized in comparison with the performance achieved using the all the TFFs extracted from the EEG channels of $G_{1}$, for both SDTP and SITP. Similarly, for amputated subjects, the results in Table 6 show that the average accuracy of the hierarchical classification framework using the TFF of $C_{1}$ has increased significantly compared with the performance achieved using all the TFFs extracted from the EEG channels of $G_{1}$, as shown in Table 5, for both SDTP and SITP. The results of the TFF-based analyses indicate that the use of more TFFs does not necessarily improve the classification accuracy. In fact, this finding can be attributed to the fact that utilizing large sets of TFFs, without applying any selection procedure, can degrade the performance of the classification nodes by exposing them to an extended group of unrelated features. Moreover, the TFF-based analyses suggest that the TFF of $C_{1}$ provides a low dimensional descriptor that can capture the intra- and inter-personal variations in the EEG signals associated with different HMITs for both intact and amputated subjects.

Furthermore, the results of the TFF-based analyses indicate that the proposed approach is significantly degraded for the SITP compared to the SDTP. This is mainly due to the large variations in the EEG signals of various HMITs across different subjects. Despite the reduction in the accuracies, the performance of the proposed approach based on the SITP is still significantly higher than the average random classification accuracy, which suggests the feasibility of applying the proposed approach to optimize the number of training sessions required to control neural prosthetic devices.

### 4.3. Comparison with Previous Approaches

Literature reveals that the majority of the existing studies have focused on classifying MI tasks associated with different limbs, such as feet, right hand, and left hand [8]. Despite the high classification accuracies attained for classifying MI tasks performed by different limbs, discriminating between the MI tasks within the same hand remains challenging [7,8,9].

Recently, few studies have investigated the possibility of applying EEG signal analysis to classify different MI tasks and actual movements within the same hand. In this vein, few researchers have investigated the possibility of decoding actual movements performed by each finger in one hand using EEG signals. For example, Liao et al. [6] proposed a pairwise binary classification scheme to discriminate between the flexion/extension movements of each pair of fingers. In particular, EEG signals were acquired from eleven right-handed intact subjects while performing flextion/extension movements of each finger using 128 electrodes. The acquired EEG signals were processed using a power spectrum decoupling procedure and principle component analysis to extract a set of features. For each pair of fingers, a SVM classifier is constructed based on the extracted features. Moreover, the training and validation of the classifier was performed using a subject-dependent scheme. In other words, for each individual subject, the SVM was trained and validated using the EEG signals of the subject under consideration without considering the EEG signals of the other subjects. The average classification accuracy computed for all pairs of fingers over all subjects was 77.1%. In comparison with the study presented in [6], our proposed hierarchial classification model incorporates a multi-class classification node, namely $CN_{6}$, that is responsible for discriminating between the flexion/extension MI tasks of the individual fingers within the same hand. In fact, Liao et al. [6] have suggested that the use of the multi-class classification scheme, which is employed in the present study, is more challenging than the pairwise classification approach that has been adopted in their study. One advantage of using the multi-class classification scheme compared to the pairwise classification is the capability to control prosthetic hands using brain signals for performing real-world tasks [6]. In terms of classification performance, $CN_{6}$ of our proposed approach has enabled the discrimination between the fingers flexion/extension MI tasks of the intact subjects with an average classification accuracies of 85.5% and 74.4% for SDTP and SITP, respectively, based on the TFF of $C_{1}$. Similarly, for amputated subjects, $CN_{6}$ achieved an average classification accuracies of 88.3% and 85.5% for SDTP and SITP, respectively. Therefore, the results of our proposed approach is considered an improvement over those reported in [6].

In another related study, Quandt et al. [33] proposed a one-versus-all multi-class classification scheme to discriminate between the movements of four fingers, which are thumb, index, middle, and little fingers. In particular, 32 EEG electrodes were utilized to record EEG signals from thirteen intact subjects while pressing and releasing a button using each of the four fingers. Then, a low-pass digital filter with bandwidth 0.15 Hz to 16 Hz was applied to the EEG signals. The samples of the filtered EEG signals were directly used as features without employing any feature selection technique. For each subject, four linear SVM classifiers were constructed using the extracted features to discriminate between the movements of the four fingers. The accuracies of classifying the movements of the thumb versus all other fingers, index versus all other fingers, middle versus all other fingers, and little versus all other fingers, which were computed for each subject individually and averaged over all subjects, were equal to 54%, 42%, 35% and 43%, respectively. Moreover, the average classification accuracy computed over these four finger movements was 43.5%. Compared with the work of Quandt et al. [33], Figure 6a shows that $CN_{6}$ of our proposed approach was able to discriminate between the flextion/extension MI tasks performed by the index, middle, ring, little, and thumb fingers with an average $F_{1}$-score of 82.8%, 88.6%, 86.1%, 84.5% and 85.1%, respectively, using the SDTP. These results indicate that our proposed approach is an improvement over the work presented in [33], taking into consideration that our proposed approach classifies various types of MI tasks besides the fingers flexion/extension imagery tasks.

Other groups of researchers focused on investigating the possibility of classifying different wrist MI movements within the same hand using EEG signals. In this vein, Vuckovic and Sepulveda [5] developed an EEG-based BCI system to classify four different wrist MI movements, including flexion, extension, pronation, and supination. Specifically, a pairwise binary classification scheme was used to differentiate between the four wrist imagery movements, such that one binary classifier was employed to classify each pair of wrist movements. To evaluate the performance of the BCI system, EEG signals were recorded for ten intact subjects using 64 electrodes. Then, feature extraction and selection were performed using Gabor transform and Davis-Douldin index methods, respectively. For each subject, an Elman recurrent neural network was constructed using the extracted features to classify between each pair of wrist imagery movements. The classification results reported in [5] indicate that the true positive rate was as high as 80%. In a relevant study, Edelman et al. [9] utilized an EEG source imaging (ESI) technique to classify four wrist MI movements associated with the right hand, including flexion, extension, supination, and pronation. The EEG signals were recorded using 64 EEG channels for five intact subjects. The EEG signals were processed using wavelet-based time-frequency analysis and Mahalanobis distance (MD)-based multi-class classifier to differentiate between the four wrist MI movements. The average classification accuracy was 82.2%.

In comparison with the studies presented in [5,9], for the intact subjects, Table 4 indicates that using any of the five TFFs categories, our proposed approach outperforms the results reported in [5,9]. For example, using the TFFs of $C_{3}$, $CN_{5}$ was able to discriminate between the wrist flexion/extension and wrist ulnar/radial deviation MI tasks with average classification accuracies of 84.4% and 60.3% for SDTP and SITP, respectively. Similarly, using the TFF of $C_{1}$, $CN_{5}$ was able to discriminate between the wrist flexion/extension and wrist ulnar/radial deviation MI tasks with average classification accuracies of 94.9% and 86.9% for SDTP and SITP, respectively.

In another study, Yong and Menon [8] proposed an EEG-based BCI system that can discriminate between four MI tasks within the same limb, including rest, grasp movement, elbow movements, and goal-oriented elbow movements. EEG signals were recorded for twelve intact subjects using 32 electrodes. Several configurations of feature extraction and classification methods were evaluated, and the best classification accuracies were achieved using the logarithmic band-power feature extraction method and the SVM classifier. The training of the SVM classifier was performed using a subject-dependent procedure. The average classification accuracies reported for the differentiation between rest versus grasp, rest versus elbow, and rest versus goal-oriented elbow movements were 80.5%, 75.1% and 76.6%, respectively. Moreover, the average three-way classification accuracies in discriminating between rest, grasp, and elbow was 56.2% and between rest, grasp, and goal-oriented elbow was 60.7%. In comparison, the $CN_{1}$ of our proposed approach was able to discriminate between the rest configuration of the hand and the 10 different HMITs of the intact subjects with average classification accuracies of 85.1% and 76.6% for SDTP and SITP, respectively, using the TFF of $C_{1}$. Furthermore, using the TFF of $C_{1}$, $CN_{3}$ was able to discriminate between three task-oriented MI grasp-related movements, namely small diameter grasp, lateral grasp, and extension-type grasp, which were performed by the intact subjects, with average classification accuracies of 87.6% and 80.8% for SDTP and SITP, respectively. Therefore, the results of our proposed approach show an improvement over the work presented in [8] with respect to the number of HMITs being classified and the ability to generalize to new subjects.

## 5. Conclusions and Future Work

In this paper, we investigated the capability for decoding eleven MI tasks within the same hand using EEG signals. In particular, the CWD was employed to extract a set of TFFs from the EEG signals. The extracted TFFs are processed using a four-layer hierarchical classification model to classify each EEG segment into one of the eleven MI tasks. Two different performance evaluation analyses were conducted to quantify the effect of utilizing different combinations of EEG channels and TFFs on the capability of the proposed approach to decode MI tasks within the same hand. These performance evaluation analyses were applied to the EEG signals obtained from eighteen intact subjects and four amputated subjects. For the intact subjects, the results of the channel-based and TFF-based analyses show that the proposed system achieved average accuracies of 88.8% and 80.8% for the SDTP and SITP, respectively, using the TFF of $C_{1}$ that are extracted from the EEG channels in $G_{1}$. Similarly, for the amputated subjects, the results of the channel-based and TFF-based analyses show that our proposed system achieved average accuracies of 90.2% and 87.8% for SDTP and SITP, respectively, using the TFF of $C_{1}$ that are extracted from the EEG channels in $G_{1}$. The results reported in this study demonstrate the feasibility of utilizing the CWD as a time-frequency representation of EEG signals to enable the extraction of TFFs that can effectively discriminate between the MI tasks within the same hand for both intact and amputated subjects. Moreover, the results reported for the SITP indicate that the CWD-based TFFs capture the inter-personal variations in the EEG signals for different HMITs. In fact, such a capability can increase the control dimension of EEG-based BCI systems to better control dexterous prosthetic hands.

Although the main goal of our proposed approach is to investigate the possibility of classifying MI tasks within the same hand using EEG signals, the current study did not consider the problem of describing the kinematic information of the MI tasks based on EEG signals. The kinematic information of the hand’s joints, such as the fingers and wrist joint angles, is important to effectively control the prosthetic hand in performing dexterous tasks. Therefore, we believe that combining the MI tasks classification, which is considered in the current study, and the hand kinematic information, which we plan to investigate in the near future, would enable better real-time control of prosthetic hands. Moreover, our future research directions include comparing the performance of our proposed approach in recognizing different MI tasks based on EEG signals that are acquired from closed-eyes subjects with the performance achieved when the eyes are open. Such a comparison can be of great benefit to assess the performance of real-world applications that involve subjects controlling prosthetic hands while their eyes are open.

## Figures and Tables

**Table 1:** Detailed information about the amputation associated with each subject in $DB_{2}$.

Subject	Handedness	Amputated Hand	Years Since Amputation	Cause of Amputation	Prosthesis Use
$AS_{1}$	Right hand	Left hand	3.5	Accident	Cosmetic
$AS_{2}$	Right hand	Right hand	1.5	Accident	None
$AS_{3}$	Left hand	Left hand	4	Accident	Myoelectric
$AS_{4}$	Right hand	Right hand	5	Accident	Cosmetic

**Table 2:** The groups of electrodes analyzed in this study.

Group Name	Comprised Electrodes
Broad bilateral ($G_{1}$)	$C_{3}$, $C_{4}$, $C_{z}$, $P_{3}$, $P_{4}$, $P_{z}$, $F_{3}$, $F_{4}$, $F_{z}$, $T_{7}$, $T_{8}$
Left side ($G_{2}$)	$C_{3}$, $P_{3}$, $F_{3}$, $T_{7}$
Right side ($G_{3}$)	$C_{4}$, $P_{4}$, $F_{4}$, $T_{8}$
Narrow bilateral ($G_{4}$)	$C_{3}$, $C_{4}$, $C_{z}$, $P_{z}$, $F_{z}$

**Table 3:** Results of the channel-based analysis obtained using the SDTP and SITP for the intact subjects in $DB_{1}$. The set of EEG electrodes comprised in each group is provided in Table 2.

Group of EEG Electrodes	Classification Layer	Classification Node	Results of the SDTP				Results of the SITP			
			PRC	RCL	$F_{1}$-Score	ACC	PRC	RCL	$F_{1}$-Score	ACC
$G_{1}$	Layer 1	$CN_{1}$	81.0	78.3	79.6	82.9	74.1	71.7	72.9	76.0
	Layer 2	$CN_{2}$	83.5	82.3	82.9	83.7	66.5	65.4	65.9	72.0
	Layer 3	$CN_{3}$	86.5	86.0	86.2	85.8	58.7	58.5	58.6	60.9
		$CN_{4}$	86.8	83.2	85.0	87.8	71.5	63.4	67.2	76.2
	Layer 4	$CN_{5}$	91.0	90.9	91.0	90.7	68.8	68.7	68.7	69.7
		$CN_{6}$	77.9	76.6	77.2	76.9	45.6	45.5	45.6	51.9
	**Overall average**		**84.4**	**82.9**	**83.6**	**84.6**	**64.2**	**62.2**	**63.2**	**67.8**
$G_{2}$	Layer 1	$CN_{1}$	77.4	72.1	74.7	77.2	70.3	67.0	68.6	73.1
	Layer 2	$CN_{2}$	73.3	72.3	72.8	74.4	65.3	58.4	61.6	65.8
	Layer 3	$CN_{3}$	74.2	73.7	73.9	73.6	49.3	49.4	49.4	49.3
		$CN_{4}$	76.7	69.0	72.6	79.1	70.1	52.5	60.1	72.8
	Layer 4	$CN_{5}$	84.1	83.3	83.7	83.7	65.5	65.5	65.5	65.5
		$CN_{6}$	63.7	62.6	63.2	62.9	37.6	37.0	37.3	37.8
	**Overall average**		**74.9**	**72.2**	**73.5**	**75.1**	**59.7**	**55.0**	**57.1**	**60.7**
$G_{3}$	Layer 1	$CN_{1}$	76.6	74.7	75.6	78.6	70.7	68.0	69.3	73.2
	Layer 2	$CN_{2}$	73.0	71.7	72.3	73.7	61.9	57.2	59.5	63.6
	Layer 3	$CN_{3}$	69.3	69.0	69.2	69.0	46.8	46.4	46.6	47.1
		$CN_{4}$	76.9	71.1	73.9	79.0	70.3	51.6	59.5	72.7
	Layer 4	$CN_{5}$	83.9	83.1	83.5	83.2	60.4	60.5	60.4	60.4
		$CN_{6}$	59.6	58.6	59.1	59.4	34.4	34.1	34.3	35.0
	**Overall average**		**73.2**	**71.4**	**72.3**	**73.8**	**57.4**	**53.0**	**54.9**	**58.7**
$G_{4}$	Layer 1	$CN_{1}$	75.6	72.1	73.8	76.6	67.3	64.4	65.8	70.1
	Layer 2	$CN_{2}$	73.5	73.1	73.3	74.5	64.6	57.1	60.6	65.5
	Layer 3	$CN_{3}$	71.7	71.3	71.5	70.8	49.6	49.3	49.5	50.1
		$CN_{4}$	78.5	70.8	74.5	80.1	70.2	52.9	60.3	71.2
	Layer 4	$CN_{5}$	83.1	82.9	83.0	82.9	69.6	69.5	69.5	66.5
		$CN_{6}$	62.0	61.7	61.9	61.5	36.6	36.1	36.3	37.0
	**Overall average**		**74.1**	**72.0**	**73.0**	**74.4**	**59.6**	**54.9**	**57.0**	**60.1**

**Table 4:** Results of the TFF-based analysis obtained using the SDTP and SITP for the intact subjects in $DB_{1}$.

Category of TFFs	Classification Layer	Classification Node	Results of the SDTP				Results of the SITP			
			PRC	RCL	$F_{1}$-Score	ACC	PRC	RCL	$F_{1}$-Score	ACC
**Log-amplitude-based** **category ($C_{1}$)**	Layer 1	$CN_{1}$	81.4	77.1	79.2	85.1	77.4	72.6	74.9	76.6
	Layer 2	$CN_{2}$	87.6	86.8	87.2	87.8	82.6	81.8	82.2	81.7
	Layer 3	$CN_{3}$	88.4	88.1	88.3	87.6	81.9	80.8	81.3	80.8
		$CN_{4}$	91.0	87.8	89.3	91.7	83.0	80.6	81.8	84.6
	Layer 4	$CN_{5}$	95.1	94.7	94.9	94.9	86.9	85.9	86.3	86.9
		$CN_{6}$	85.0	86.0	85.5	85.5	76.2	75.1	75.6	74.4
	**Overall average**		**88.1**	**86.7**	**87.4**	**88.8**	**81.3**	**79.5**	**80.4**	**80.8**
**Amplitude-based** **category ($C_{2}$)**	Layer 1	$CN_{1}$	78.8	76.3	77.5	80.5	72.7	69.2	70.9	70.0
	Layer 2	$CN_{2}$	79.6	78.3	78.9	80.2	72.2	70.0	71.1	62.1
	Layer 3	$CN_{3}$	81.3	80.7	81.0	80.3	67.0	65.3	66.1	61.2
		$CN_{4}$	84.9	80.4	82.6	86.9	71.9	62.6	66.9	70.9
	Layer 4	$CN_{5}$	89.6	89.9	89.7	89.8	71.7	70.4	71.1	68.4
		$CN_{6}$	71.2	71.0	71.1	71.5	59.8	56.2	57.9	53.4
	**Overall average**		**80.9**	**79.4**	**80.1**	**81.5**	**69.2**	**65.6**	**67.4**	**64.3**
**Statistical-based** **category ($C_{3}$)**	Layer 1	$CN_{1}$	76.4	73.2	74.8	77.6	67.2	62.7	64.9	65.9
	Layer 2	$CN_{2}$	74.2	72.9	73.5	75.2	59.8	55.1	57.3	56.5
	Layer 3	$CN_{3}$	72.7	72.6	72.6	72.5	46.3	46.0	46.2	51.5
		$CN_{4}$	75.7	71.6	73.6	79.3	65.8	52.4	58.3	57.7
	Layer 4	$CN_{5}$	84.4	85.0	84.7	84.4	61.4	61.4	61.4	60.3
		$CN_{6}$	63.1	62.2	62.7	62.5	31.7	30.3	31.0	40.8
	**Overall average**		**74.4**	**72.9**	**73.6**	**75.2**	**55.4**	**51.3**	**53.2**	**55.4**
**Spectral-based** **category ($C_{4}$)**	Layer 1	$CN_{1}$	79.5	80.3	79.9	83.1	72.9	70.1	71.5	72.3
	Layer 2	$CN_{2}$	85.4	85.0	85.2	86.0	73.5	72.6	73.0	71.1
	Layer 3	$CN_{3}$	87.3	87.3	87.3	86.8	69.6	69.4	69.5	66.1
		$CN_{4}$	89.1	87.7	88.4	90.7	72.3	67.4	69.8	72.6
	Layer 4	$CN_{5}$	91.3	90.6	91.0	92.8	80.4	80.3	80.3	79.1
		$CN_{6}$	81.9	82.4	82.1	82.4	60.3	59.4	59.9	57.8
	**Overall average**		**85.7**	**85.5**	**85.6**	**87.0**	**71.5**	**69.9**	**70.7**	**69.8**
**Spectral entropy-based** **category ($C_{5}$)**	Layer 1	$CN_{1}$	78.0	76.5	77.2	80.1	70.3	65.8	68.0	69.6
	Layer 2	$CN_{2}$	80.3	79.7	80.0	81.1	63.6	60.0	61.7	63.0
	Layer 3	$CN_{3}$	81.7	81.3	81.5	81.1	56.0	56.0	56.0	55.4
		$CN_{4}$	84.5	81.8	83.1	86.6	63.1	56.7	59.7	76.5
	Layer 4	$CN_{5}$	89.4	89.8	89.6	89.6	69.1	69.1	69.1	70.5
		$CN_{6}$	73.5	74.6	74.0	73.9	37.7	37.3	37.5	40.5
	**Overall average**		**81.2**	**80.6**	**80.9**	**82.1**	**60.0**	**57.5**	**58.7**	**62.6**

**Table 5:** Results of the channel-based analysis obtained using the SDTP and SITP for the amputated subjects in $DB_{2}$.

Group of EEG Electrodes	Classification Layer	Classification Node	Results of SDTP				Results of SITP			
			PRC	RCL	$F_{1}$-Score	ACC	PRC	RCL	$F_{1}$-Score	ACC
$G_{1}$	Layer 1	$CN_{1}$	79.7	77.3	78.5	80.5	74.3	72.7	73.5	75.1
	Layer 2	$CN_{2}$	81.5	79.3	80.4	81.5	69.8	68.0	68.9	70.8
	Layer 3	$CN_{3}$	85.2	84.6	84.9	84.6	76.8	76.5	76.6	76.4
		$CN_{4}$	88.8	86.0	87.3	89.1	77.0	74.9	75.9	81.7
	Layer 4	$CN_{5}$	92.1	92.3	92.2	92.2	82.7	82.3	82.5	82.0
		$CN_{6}$	81.3	81.4	81.4	81.2	57.4	57.6	57.5	57.5
	**Overall average**		**84.8**	**83.5**	**84.1**	**84.8**	**73.0**	**72.0**	**72.5**	**73.9**
$G_{2}$	Layer 1	$CN_{1}$	76.7	74.9	75.8	77.7	66.6	64.9	65.8	71.4
	Layer 2	$CN_{2}$	77.5	76.8	77.2	78.4	65.0	58.9	61.8	64.0
	Layer 3	$CN_{3}$	83.9	81.7	82.7	81.2	58.0	57.8	57.9	58.1
		$CN_{4}$	86.5	83.8	85.1	85.3	67.6	63.9	65.7	65.0
	Layer 4	$CN_{5}$	85.0	85.8	85.4	84.7	77.7	78.1	77.9	77.9
		$CN_{6}$	72.1	72.6	72.3	73.5	45.7	44.4	45.0	45.6
	**Overall average**		**80.3**	**79.3**	**79.8**	**80.2**	**63.4**	**61.3**	**62.3**	**63.6**
$G_{3}$	Layer 1	$CN_{1}$	77.0	75.9	76.5	78.7	68.4	67.3	67.8	70.0
	Layer 2	$CN_{2}$	79.7	78.3	79.0	80.4	66.7	63.8	65.2	67.6
	Layer 3	$CN_{3}$	85.2	83.3	84.3	83.8	62.2	62.4	62.3	62.2
		$CN_{4}$	85.5	84.4	85.0	85.2	70.0	59.0	64.0	67.6
	Layer 4	$CN_{5}$	87.4	86.3	86.9	85.0	75.5	75.4	75.5	75.4
		$CN_{6}$	76.4	76.8	76.6	76.4	46.5	46.1	46.3	46.9
	**Overall average**		**81.9**	**80.9**	**81.4**	**81.6**	**64.9**	**62.3**	**63.5**	**64.9**
$G_{4}$	Layer 1	$CN_{1}$	76.7	74.4	75.6	75.2	66.7	62.0	64.3	67.6
	Layer 2	$CN_{2}$	80.7	79.1	79.9	80.7	66.2	62.5	64.3	67.7
	Layer 3	$CN_{3}$	84.3	81.3	82.8	82.7	69.8	69.9	69.8	67.0
		$CN_{4}$	87.9	86.8	87.3	87.3	75.5	68.3	71.7	79.8
	Layer 4	$CN_{5}$	90.5	90.7	90.6	89.1	70.6	70.3	70.5	70.5
		$CN_{6}$	78.1	77.1	77.6	77.7	53.0	52.1	52.5	52.2
	**Overall average**		**83.0**	**81.6**	**82.3**	**82.1**	**67.0**	**64.2**	**65.5**	**67.5**

**Table 6:** Results of the TFF-based analysis obtained using the SDTP and SITP for the amputated subjects in $DB_{2}$.

Category of TFFs	Classification Layer	Classification Node	Results of the SDTP				Results of the SITP			
			PRC	RCL	$F_{1}$-Score	ACC	PRC	RCL	$F_{1}$-Score	ACC
**Log-amplitude-based** **category ($C_{1}$)**	Layer 1	$CN_{1}$	79.3	78.0	78.6	77.8	76.5	74.8	75.6	77.6
	Layer 2	$CN_{2}$	90.7	88.6	89.7	90.0	88.8	88.4	88.6	89.1
	Layer 3	$CN_{3}$	93.0	93.0	93.0	93.1	90.7	90.5	90.6	90.6
		$CN_{4}$	93.6	91.7	92.7	94.6	92.4	85.5	88.8	90.5
	Layer 4	$CN_{5}$	97.4	97.3	97.4	97.4	93.3	93.5	93.4	93.4
		$CN_{6}$	89.3	88.4	88.8	88.3	86.0	85.6	85.8	85.5
	**Overall average**		**90.5**	**89.5**	**90.0**	**90.2**	**87.9**	**86.4**	**87.1**	**87.8**
**Amplitude-based** **category ($C_{2}$)**	Layer 1	$CN_{1}$	72.3	71.6	72.0	74.8	71.8	69.0	70.4	74.0
	Layer 2	$CN_{2}$	84.7	82.3	83.5	84.0	77.6	73.9	75.7	76.7
	Layer 3	$CN_{3}$	84.8	83.4	84.1	83.8	73.6	72.8	73.2	73.0
		$CN_{4}$	91.3	86.8	89.0	90.7	87.1	78.5	82.6	85.7
	Layer 4	$CN_{5}$	85.3	84.9	85.1	84.9	85.4	86.2	85.8	85.2
		$CN_{6}$	72.7	72.8	72.8	72.3	62.9	61.7	62.3	61.6
	**Overall average**		**81.8**	**80.3**	**81.1**	**81.7**	**76.4**	**73.7**	**75.0**	**76.0**
**Statistical-based** **category ($C_{3}$)**	Layer 1	$CN_{1}$	71.2	68.7	69.9	73.7	67.0	63.1	65.0	68.2
	Layer 2	$CN_{2}$	72.1	70.6	71.4	73.6	61.7	57.4	59.4	63.0
	Layer 3	$CN_{3}$	74.4	74.0	74.2	74.6	52.6	51.4	52.0	51.3
		$CN_{4}$	78.7	78.6	78.7	82.9	67.7	63.3	65.4	74.0
	Layer 4	$CN_{5}$	83.9	83.4	83.6	83.9	72.5	73.0	72.8	72.1
		$CN_{6}$	60.8	60.3	60.5	60.5	38.5	36.1	37.3	37.1
	**Overall average**		**73.5**	**72.6**	**73.1**	**74.9**	**60.0**	**57.4**	**58.7**	**61.0**
**Spectral-based** **category ($C_{4}$)**	Layer 1	$CN_{1}$	76.4	75.1	75.7	75.6	74.4	70.8	72.6	74.7
	Layer 2	$CN_{2}$	85.6	84.5	85.0	85.5	77.8	76.8	77.3	78.3
	Layer 3	$CN_{3}$	92.1	91.5	91.8	91.9	83.8	82.9	83.3	82.8
		$CN_{4}$	92.7	91.8	92.3	93.1	85.1	76.9	80.8	85.0
	Layer 4	$CN_{5}$	92.7	92.7	92.7	92.8	87.4	87.6	87.5	87.7
		$CN_{6}$	87.5	86.5	87.0	86.9	70.2	69.9	70.1	70.4
	**Overall average**		**87.8**	**87.0**	**87.4**	**87.6**	**79.8**	**77.5**	**78.6**	**79.8**
**Spectral entropy-based** **category ($C_{5}$)**	Layer 1	$CN_{1}$	75.4	72.3	73.8	77.5	69.1	66.7	67.9	71.8
	Layer 2	$CN_{2}$	76.7	75.4	76.1	77.6	65.4	63.0	64.2	66.4
	Layer 3	$CN_{3}$	86.3	85.8	86.1	86.4	67.9	66.9	67.4	67.8
		$CN_{4}$	87.4	84.5	85.9	88.6	76.0	73.3	74.6	79.3
	Layer 4	$CN_{5}$	88.8	89.3	89.0	88.6	82.4	82.3	82.4	82.8
		$CN_{6}$	77.2	76.7	77.0	76.4	51.3	51.0	51.2	51.3
	**Overall average**		**82.0**	**80.7**	**81.3**	**82.5**	**68.7**	**67.2**	**67.9**	**69.9**
